# Electrical Performance and Reliability Improvement of Amorphous-Indium-Gallium-Zinc-Oxide Thin-Film Transistors with HfO_2_ Gate Dielectrics by CF_4_ Plasma Treatment

**DOI:** 10.3390/ma11050824

**Published:** 2018-05-17

**Authors:** Ching-Lin Fan, Fan-Ping Tseng, Chiao-Yuan Tseng

**Affiliations:** 1 Department of Electronic Engineering, National Taiwan University of Science and Technology, 43 Section 4, Keelung Road, Taipei 106, Taiwan; m10402321@mail.ntust.edu.tw; 2 Graduate Institute of Electro-Optical Engineering, National Taiwan University of Science and Technology, 43 Section 4, Keelung Road, Taipei 106, Taiwan; d10219001@mail.ntust.edu.tw

**Keywords:** a-IGZO TFT, HfO_2_ gate dielectric, plasma treatment, fluorine, reliability

## Abstract

In this work, amorphous indium-gallium-zinc oxide thin-film transistors (a-IGZO TFTs) with a HfO_2_ gate insulator and CF_4_ plasma treatment was demonstrated for the first time. Through the plasma treatment, both the electrical performance and reliability of the a-IGZO TFT with HfO_2_ gate dielectric were improved. The carrier mobility significantly increased by 80.8%, from 30.2 cm^2^/V∙s (without treatment) to 54.6 cm^2^/V∙s (with CF_4_ plasma treatment), which is due to the incorporated fluorine not only providing an extra electron to the IGZO, but also passivating the interface trap density. In addition, the reliability of the a-IGZO TFT with HfO_2_ gate dielectric has also been improved by the CF_4_ plasma treatment. By applying the CF_4_ plasma treatment to the a-IGZO TFT, the hysteresis effect of the device has been improved and the device’s immunity against moisture from the ambient atmosphere has been enhanced. It is believed that the CF_4_ plasma treatment not only significantly improves the electrical performance of a-IGZO TFT with HfO_2_ gate dielectric, but also enhances the device’s reliability.

## 1. Introduction

Recently, amorphous indium-gallium-zinc oxide thin-film transistors (a-IGZO TFT) have attracted considerable attention in flat-panel displays (FPDs), because of its advantages, including high carrier mobility (>10 cm^2^/V∙s), good uniformity in large-area deposition and low temperature fabrication [1,2,3,4,5]. Compared with low-temperature poly-crystalline silicon (LTPS) TFTs, the a-IGZO TFTs are more suitable to utilize as driving-TFTs in large-area active-matrix organic light-emitting diode (AM-OLED) displays because of these aforementioned advantages. The a-IGZO TFT is a good candidate to replace silicon-based TFTs both in the active-matrix liquid crystal displays (AM-LCDs) and AM-OLED displays.

In order to increase the carrier mobility of the a-IGZO TFT, many previous researchers used a high dielectric constant (high-k) material as the gate insulator to improve the electrical performance of a-IGZO TFT [6,7,8,9,10,11]. Among these gate insulator materials, HfO_2_ is one of the most promising high-k materials due to its advantages of a high dielectric constant (k > 20), a sufficient energy bandgap offset and a suitable interface for the IGZO semiconductor [12,13,14,15,16,17]. However, the electrical stability of the a-IGZO TFT with a high-k gate dielectric layer is one of the critical issues, especially under the positive-gate bias stress (PGBS) [10]. The threshold voltage (V_th_) is abnormally shifted in the negative direction, which is opposite to the a-IGZO TFT with a SiO_2_ gate insulator [18,19]. This is due to the electron density generated by the density of states (DOS) near the dielectric/channel layer interface or from absorbed moisture from the air. In addition, the higher dielectric constant of the high-k gate insulator would induce a higher electric-field through the active layer, which makes the a-IGZO TFTs more sensitive to the ambient molecules [20]. Therefore, the reliability of the a-IGZO TFT with high-k gate dielectric under PGBS and under ambient atmosphere should be enhanced.

It is reported that fluorine incorporated into the a-IGZO TFT can effectively improve TFT reliability [20,21,22,23,24,25,26], because F has several advantages: (i) it has the highest electron affinity among chemical elements, which makes F simple to bond with misoriented metal atoms in the IGZO active layer and reduce the DOS, (ii) compared to hydrogen, F can provide stronger bonding with the metal ion in the active layer to improve the a-IGZO TFT stability [27], and (iii) extra free electrons can be generated by the fluorine ions by replacing the oxygen sites, due to the difference in electrovalence between the oxygen ion (O^2−^) and the fluorine ion (F^−^). The higher electron density induced in the active layer will improve the carrier mobility of the a-IGZO TFT [28]. Recently, some researchers reported using carbon fluoride mixed with oxygen gas (CF_4_/O_2_ and CHF_3_/O_2_) to enhance the stability of a-IGZO TFT [20,21,22]. Although the stability under the PGBS of the a-IGZO TFT was enhanced, the electrical performance of the TFT was not significantly improved. It is well-known that the oxygen molecule acts as a carrier suppressor in the IGZO thin film. The merit of F ions that can generate extra electrons in the IGZO film might be diluted by the incorporation of oxygen. In addition, there is no research that has focused on the effects of plasma treatment in the a-IGZO TFT with the HfO_2_ material as the gate insulator. The a-IGZO TFT with the HfO_2_ gate insulator and treatment by CF_4_ plasma is studied here for the first time.

In this work, both the electrical performance and reliability of the a-IGZO TFTs were improved by the CF_4_ plasma treatment, and with HfO_2_ as the gate insulator. After the CF_4_ plasma treatment of the active layer, the carrier mobility significantly improved from 30.2 cm^2^/V∙s to 54.6 cm^2^/V∙s. The improvement is attributed to the extra electrons generated in the IGZO film and the DOS near the gate insulator/channel interface passivated by the incorporation of fluorine. The effect of the reduced DOS near the interface also can be distinctly observed in the hysteresis measurement. In addition, the moisture absorption effect was also investigated in this work. It was found that the CF_4_ plasma treated a-IGZO TFT has higher immunity against ambient moisture.

## 2. Device Fabrication 

**Figure 1:**
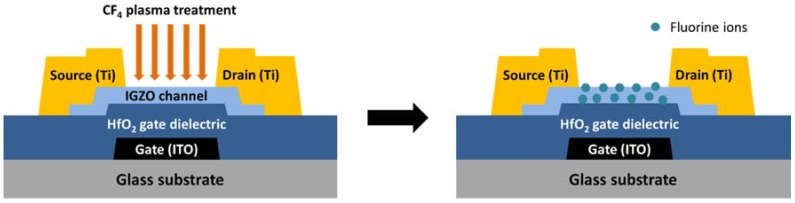
Schematic cross-sectional diagrams of a-IGZO TFT with CF_4_ plasma treatment.

Figure 1 shows the schematic cross-sectional diagrams of the bottom-gate, top-contact a-IGZO TFTs. First, ITO film was patterned using photolithography and wet etching to form the gate electrode. The 180-nm thick HfO_2_ was then deposited as the gate insulator layer by RF sputtering in the mixed gas O_2_/Ar = 33% and annealed at 250 °C for 60 min. After patterning the contact hole, a 30 nm a-IGZO thin-film was deposited by RF sputtering in an Ar environment at room temperature. The active layer was then patterned by wet etching. A 160 nm Ti layer was deposited by thermal evaporation and using lift-off process to form source and drain (S/D) electrodes. Finally, a CF_4_ plasma treatment was carried out at the RF power of 15 W for 20 s, followed by a post-anneal at 250 °C for 60 min. Note that the low power and short treatment time of the plasma treatment ensured that the plasma treatment mainly acted on the IGZO active layer without serious damage.

The electrical characteristics and the bias stress voltage were examined using an Agilent 4145B semiconductor parameter analyzer in the dark. The transfer curves were measured at a source-to-drain voltage (V_DS_) of 5 V and the Vth was extracted from linear extrapolations of the square root plot of the drain current (I_DS_). The channel width (W) and length (L) of the a-IGZO TFT were 50 µm and 5 µm, respectively. The a-IGZO TFT without the passivation layer was used in the positive-gate bias stress (PGBS) to examine the ambient effect. The PGBS was carried out at the gate electrode (V_GS_ = 6 V) with the source and drain electrodes grounded. 

## 3. Results and Discussion

**Figure 2:**
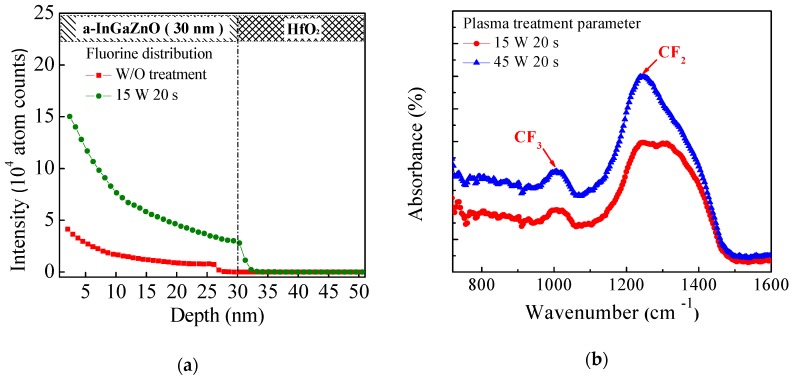
(**a**) SIMS depth profiles; (**b**) FTIR measurement for the untreated sample and the sample with the CF_4_ plasma treatment after annealing at 250 °C for 60 min.

Figure 2a shows the secondary ion mass spectrometry (SIMS) depth profile in the stack of IGZO/HfO_2_/Si thin films. Compared with the untreated sample, the higher F concentration is observed in the sample with the CF_4_ plasma treatment, which indicates that the F atoms were successfully introduced into the IGZO film by the CF_4_ plasma treatment. In addition, the F atoms diffused towards the bulk of the IGZO film, and piled up at the IGZO/HfO_2_ interface. Figure 2b shows the fourier-transform infrared spectroscopy (FTIR) measurement of the IGZO film. The wave numbers of 980 cm^−1^ and 1240 cm^−1^ refer to the CF_3_ bond and CF_2_ bond, respectively [29]. Evidently, the IGZO film with 45 W treatment power has the higher CF_2_ peak, which indicates the more serious etching effect during the plasma treatment. CF_4_ is well-known as one of the reactive etching gas used in dry etching process. As the RF power increased, CF_4_ begins to dissociate into smaller components, such as CF_2_, CF_3_ and F radicals, which etch the underlying IGZO thin film. A small treatment power can reduce plasma damage in the IGZO active layer.

**Figure 3:**
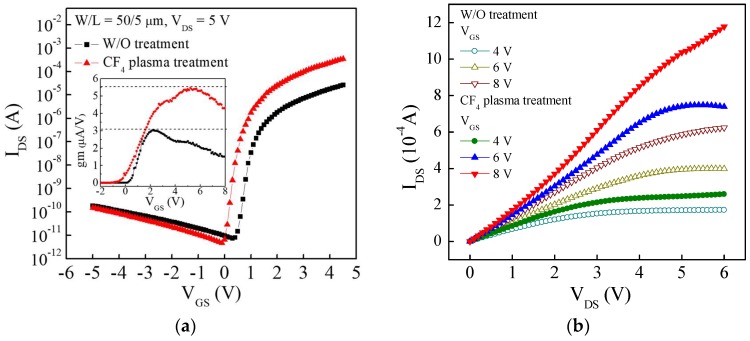
(**a**) Transfer curves (I_DS_-V_GS_); (**b**) Output curves (I_DS_-V_DS_) of the a-IGZO TFT without treatment and with a 15 W, 20 s CF_4_ plasma treatment. Inset of Figure 3a shows the transconductance measured at V_DS_ = 0.1 V.

Figure 3a shows the transfer curves of the a-IGZO TFT with and without the CF_4_ plasma treatment. The linear mobility (µ_linear_), maximum transconductance (gm_max_), threshold voltage (V_th_), subthreshold swing (S.S.) and the on/off current ratio (I_on_/I_off_) are summarized in Table 1. The µ_linear_ is calculated from the gm_max_ measured at the V_DS_ = 0.1 V using the linear-region drain current function, which is shown in the inset of Figure 3a. It is obvious that the a-IGZO TFT with the CF_4_ plasma treatment has a higher gm_max_ compared to the TFT without treatment, and that the linear mobility of the device is significantly improved from 30.2 cm^2^/V∙s (without treatment) to 54.6 cm^2^/V∙s (with CF_4_ plasma treatment). Moreover, the V_th_ and S.S. were slightly decreased from 1.50 V to 1.05 V, and 0.17 V/decade to 0.14 V/decade, respectively. The carrier mobility of TFT mainly depended on the carrier scattering or trapping in the channel layer. It is reasonable to suggest that the carrier concentration and trap density will dominate the carrier mobility. It is important to consider that the role of F atom in the IGZO film can not only passivate the defects by replacing the weakly bonded oxygen atoms or by directly filling the oxygen vacancy, but will also donate extra electrons due to the difference in electrovalence between the oxygen ion (O^2−^) and the fluorine ion (F^−^) [28]. A lower interface trap density results in less carrier scattering, and the gate bias voltage can effectively induce more carrier concentration to transport in the IGZO channel due to the decreased trap density and the donated extra electrons. As a result, the carrier mobility of the a-IGZO TFT is improved by the CF_4_ plasma treatment. Figure 3b shows the output curves of the two a-IGZO TFTs measured at V_GS_ = 4 V, 6 V, and 8 V. Both the TFTs exhibit n-type enhancement mode, and the I_DS_ saturated at the high V_DS_ region. Compared with the untreated device, the higher I_DS_ observed in the a-IGZO TFT with the CF_4_ plasma treatment resulted from the increased carrier mobility. The on currents measured at V_DS_ = 6 V and V_GS_ = 8 V of the plasma treatment TFT and the untreated TFT are 1180 µA and 623 µA, respectively. As a result, the electrical performance of the a-IGZO TFT can be significantly improved by the CF_4_ plasma treatment.

**Figure 4:**
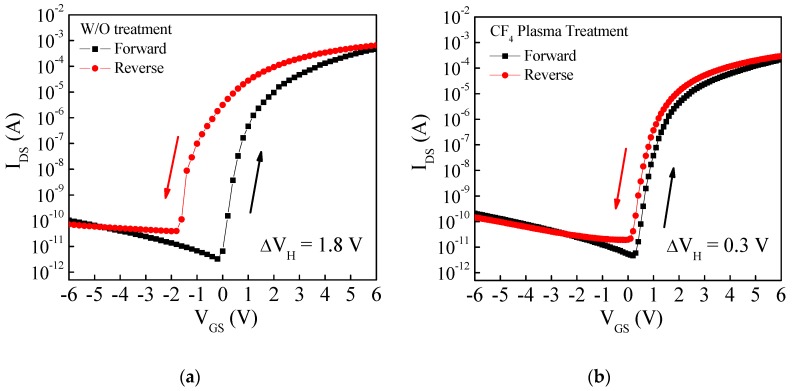
Hysteresis measurement of amorphous indium-gallium-zinc oxide (a-IGZO) thin-film transistor (TFT) with and without the CF_4_ plasma treatment.

Figure 4 shows the hysteresis measurement of the a-IGZO TFT with and without the CF_4_ plasma treatment. It can be observed that the hysteresis voltage (∆V_H_) abnormally shifted towards the negative direction and the a-IGZO TFT with the plasma treatment has a smaller ∆V_H_. The ∆V_H_ of the a-IGZO TFT with and without the CF_4_ plasma treatment are −1.8 V and −0.3 V, respectively. The negative V_th_ shift is due to the carrier creation in the IGZO film, which is mainly from the enhanced control and from using the high-k HfO_2_ as the gate insulator. When the gate bias is applied to the TFT, the HfO_2_ gate insulator will provide a strong electric field near the IGZO/HfO_2_ interface to bend the Fermi level in the deep of the band gap. The energy level of the neutral oxygen vacancies (V_O_), which is higher than the Fermi level, will be ionized (V_O_^++^) and two electrons will be contributed to the IGZO film because the highest electron energy level in the IGZO band gap cannot reach the energy level of V_O_ [10,18]. Therefore, the extra free electrons generated during the forward sweep of the gate bias will cause the negative V_th_ shift in the reverse sweep of the gate bias.

**Figure 5:**
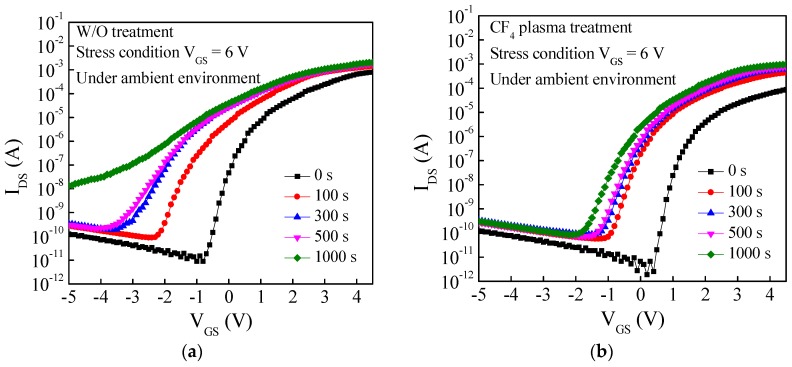
Transfer curves (I_DS_-V_GS_) of a-IGZO TFT with and without the CF_4_ plasma treatment under positive gate bias stress (PGBS) (V_GS_ = 6 V).

**Figure 6:**
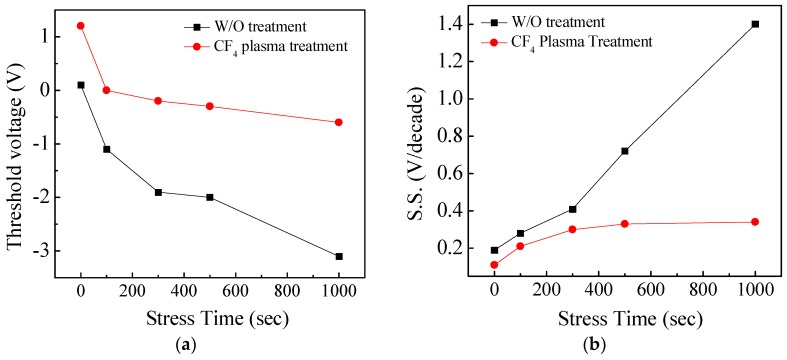
(**a**) V_th_; (**b**) S.S. as function of PGBS time of a-IGZO TFT.

Figure 5a,b shows the transfer curves of the a-IGZO TFT with and without the CF_4_ plasma treatment under the PGBS (V_GS_ = 6 V). The TFTs were fabricated without the passivation layer to examine the ambient effect. For the a-IGZO TFT without treatment, the device suffers from a more serious negative V_th_ shift and a S.S. degradation, as shown in Figure 6a,b, which are mainly due to the moisture from the ambient atmosphere absorbed onto the IGZO surface. The absorbed moisture will bond to the metal element in IGZO to form metal-hydroxide (M-OH) bonds, which act as donor-like states to provide extra electrons, but also increase the density of state within the IGZO film [18]. Thus, the absorbed moisture will cause a negative V_th_ shift and an S.S. degradation. For the a-IGZO TFT with the CF_4_ plasma treatment, the oxygen vacancies or the weak oxygen bonds on the IGZO surface are replaced by the F ions, as shown in Figure 2b. The M-F bonds are stronger and more stable than the M-O bond, and thus the formation of the M-OH bonds is suppressed by the incorporation of F. In summary, the a-IGZO TFT with HfO_2_ as the gate insulator and the CF_4_ plasma treatment not only acquires a higher immunity against humidity from ambient atmosphere, but also significantly improves the device performance.

## 4. Conclusions

We have demonstrated that the CF_4_ plasma treatment can significantly improve the electrical performance and reliability of the a-IGZO TFT with a HfO_2_ gate insulator. After the plasma treatment at 15 W for 20 s on the IGZO active layer, the carrier mobility significantly improved from 30.2 cm^2^/V∙s to 54.6 cm^2^/V∙s. This can be attributed to the incorporated F providing additional electrons in the IGZO, and in parallel passivating the interface traps at the IGZO/HfO_2_ interface. Low interface traps result in less carrier scattering, which results in an increased carrier mobility. Moreover, it was found that the CF_4_ plasma treatment can effectively suppress the hysteresis effect and enhance the device’s immunity against humidity from the ambient atmosphere. It is believed that the CF_4_ plasma treatment can improve the electrical performance and reliability of the a-IGZO TFT with a HfO_2_ gate insulator.

## Figures and Tables

**Table 1:** Effects of the CF_4_ plasma treatment on electrical performance parameters of the fabricated a-IGZO TFTs with HfO_2_ gate dielectric.

Electrical Parameters	Without Treatment	With CF_4_ Plasma Treatment
µ_linear_ (cm^2^/V∙s)	30.2	54.6
gm_max_ (A/V)	3.02 × 10^−6^	5.46 × 10^−6^
V_th_ (V)	1.50	1.05
S.S. (V/decade)	0.17	0.14
I_on_/I_off_	3.5 × 10^6^	7.44 × 10^7^
